# AARS2 as a novel biomarker for prognosis and its molecular characterization in pan‐cancer

**DOI:** 10.1002/cam4.6682

**Published:** 2023-11-21

**Authors:** Long Liu, Jie Gao, Xudong Liu, Feng Zhang, Bowen Hu, Huapeng Zhang, Zhihui Wang, Hongwei Tang, Ji Hua Shi, Shuijun Zhang

**Affiliations:** ^1^ Department of Hepatobiliary and Pancreatic Surgery The First Affiliated Hospital of Zhengzhou University Zhengzhou China; ^2^ Henan Key Laboratory for Digestive Organ Transplantation Zhengzhou China; ^3^ Open and Key Laboratory for Hepatobiliary & Pancreatic Surgery and Digestive Organ Transplantation at Henan Universities Zhengzhou China; ^4^ Henan Liver Transplantation Centre Zhengzhou China; ^5^ Henan Organ Transplantation Quality Control Centre Zhengzhou China; ^6^ Henan Research Centre for Organ Transplantation Zhengzhou China

**Keywords:** AARS2, biological function, biomarker, hepatocellular carcinoma, pan‐cancer

## Abstract

**Introduction:**

The mitochondrial alanyl‐tRNA synthetase 2 (AARS2) as one of aminoacyl‐tRNA synthases (ARSs) performs amino acid transportation and involves protein synthesis. However, its role in cancer remains largely unexplored.

**Methods:**

In this study, more than 10,000 samples were enrolled to explore genomic alterations, biological function, prognosis, and clinical treatment based on AARS2 across pan‐cancer. The molecular characterization of AARS2 was confirmed in hepatocellular carcinoma (HCC) using proteomics analysis, quantitative real‐time PCR, western blotting, immunohistochemical staining, and cell experiments.

**Results:**

For genomic landscape, the AARS2 was dramatically upregulated in multiple cancers, which might be mainly caused by copy number alteration rather than mutation and methylation. The abnormal expression of AARS2 was prominently associated with activity of cancer pathways and performed oncogenic roles in most cancers. Systematic experiments in vitro substantiated the elevated expression of AARS2, and the deficiency of it inhibited cell proliferation and cell migration in HCC. Meanwhile, our findings suggested that AARS2 could serve as a novel promising and stable biomarker for assessing prognosis and immunotherapy. Moreover, a variety of therapeutic drugs and targeted pathways were proposed for cancer treatment, which might enhance clinical efficacy.

**Conclusion:**

The AARS2 could serve as a new oncogenic gene that promotes cell proliferation and migration in HCC. The comprehensive investigations increased the understanding of AARS2 across human cancers and generated beginning insights of AARS2 in genomic landscape, molecular biological function, prognosis, and clinical treatment.

## 1 INTRODUCTION

Hepatocellular carcinoma (HCC) stands out as the most prevalent and profoundly malignant solid liver neoplasm, ranking sixth in terms of morbidity and fourth in mortality worldwide.^1^ HCC exhibits prevalent characteristics and shares hallmark features with other human cancers, undergoing a complex carcinogenic process from normalcy to neoplastic growth states.^2^ As we known, human carcinogenesis has tight links with the diversity of biological mechanisms and the variety of malignant pathogenesis.^2^, ^3^ Increasingly, studies are focusing on molecular characterization across pan‐cancer to enhance the understanding of human malignancies.^3^, ^4^, ^5^ The heat shock factor 1 (HSF1) as a latent target for prognosis and immunotherapy presents different performance in pan‐cancer, promoting oncogenesis and metastasis in some cancers (liver cancer and kidney cancer) while inhibiting oncogenesis and metastasis in some cancers (lung cancer and cholangiocarcinoma).^4^ Gasdermins (GSDMs)‐mediated pyroptosis displays tight links with accelerating anti‐tumor immunity and inhibiting tumor growth, which also harbors critical prognostic value in many types of human cancers.^5^ DNA methylation emerges as a primary indicator driving aberrant aldehyde dehydrogenase 2 (ALDH2) expression in multiple cancer contexts.^6^ For pan‐cancer explorations, more promising findings might bring novel insights into prognosis, biological behaviors, and clinical treatments. Thus, the systematic and comprehensive explorations focusing on a new molecular structure should be conducted in 33 human cancer types, increasing deeper understanding and providing effective treatment for malignancy.

Recently, the continued interest in aminoacyl‐tRNA synthases (ARSs) has delivered their pathological significance in carcinogenesis and clinical implications in therapy.^7^, ^8^ ARSs as a class of catalytic enzymes transport amino acid to cognate tRNAs for cell protein synthesis.^7^ Research has demonstrated that ARSs severed as regulators, sensing diverse cellular conditions and influencing various cellular processes, thus establishing connections with tumorigenesis.^7^ For instance, the leucine‐tRNA synthase 2 (LARS2)‐expressing B cell (LARS B) subpopulation is verified in colorectal cancer, and inhibiting LARS B cells contributes to clinical therapy.^8^ In breast cancer, the overexpression of tyrosine aminoacyl‐tRNA synthetase (YARS) could enhance chemotherapy response based on phosphorylation of necrosome complex.^9^ As one member of the ARS family, the mitochondrial alanyl‐tRNA synthetase 2 (AARS2) is coding by nuclear genome and performing function in chondriosome, which remains largely unexplored in human cancers. The AARS2 is frequently reported that its mutation has significant correlations with leukoencephalopathy.^10^ Childhood cardiomyopathies is typically caused by specific gene mutations, and the alteration of AARS2 might a crucial genetic basis.^11^ In addition, only one tumor‐associated research declares that AARS2 is a protective indicator in colorectal cancer and regulates cell proliferation via interfering mitochondrial respiration.^12^ There is a pressing need to enhance our in‐depth and comprehensive understanding of AARS2, both in terms of molecular characterization and its potential clinical implications across a wide spectrum of human cancers.

In this study, we discovered a novel tumor‐promotive factor AARS2 in HCC and decoded its molecular characterization in 33 human cancer types. Our investigation has encompassed genomic alterations, biological functions, prognostic implications, and the therapeutic potential of AARS2 in various human cancers. The copy number alteration (CNA) might be key driver to causing aberrant expression of AARS2 in many cancers. According to estimating the relationship between AARS2 and tumor biological activity and clinical transformation, the AARS2 performed as an oncogenic gene in HCC and could be a promising biomarker for prognosis and immunotherapy in human cancers. Moreover, some potential drugs and targeted pathways were identified based on AARS2 expression. Taken together, our study lays the foundation for biomarker development and clinical transformation.

## 2 METHODS

### 2.1 Sample collections and data processing

#### 2.1.1 Sample clinical information and tissue specimens

This study was performed in accordance with the Declaration of Helsinki and was approved by the Medical Ethics Committee of the First Affiliated Hospital of Zhengzhou University. According to the policies of the committee, all patients signed written informed consent. Based on histopathological HCC tissue and matched adjacent non‐tumor tissue, a total of 71 pairs of fresh tissue and 64 pairs of paraffin‐embedded specimens were collected from patients who were clinically diagnosed and underwent surgical resection at our department. All tissue specimens were confirmed by pathological diagnosis at the First Affiliated Hospital of Zhengzhou University. The clinical information of patients is available in Table S1, including age, gender, alpha fetal protein (AFP) level, greatest tumor diameter, total tumor diameter, microvascular invasion (MVI), overall survival (OS), and recurrence‐free survival (RFS). The detailed inclusion criteria and usage of specimens are referred to as supplementary Methods: Data S1.

#### 2.1.2 Public data collection and processing of 33 cancer types

A total of 33 different cancer datasets were downloaded from The Cancer Genome Atlas (TCGA, https://portal.gdc.cancer.gov/). The corresponding clinical information and CNA data were obtained from UCSC Xena portal (https://xenabrowser.net/datapages/). The expression and CNA information of human tumor cell lines were acquired from Cancer Cell Line Encyclopedia (CCLE) dataset (https://sites.broadinstitute.org/ccle). The RNA‐seq read count from the TCGA database was converted to transcripts per kilobase million (TPM) and further log‐2 transformed. In these expression matrixes, some cohorts did not contain normal samples, including ACC, DLBC, LAML, LGG, MESO, OV, TGCT, UCS, and UVM. The detailed abbreviation of 33 cancer types was displayed in Supplementary Methods: Data S1.

The genes harbored focal CNA values over 0.3 were defined as ‘gain’ (+1), and genes harbored focal CNA values less −0.3 were defined as ‘loss’ (−1). When genes harbored focal CNA values from −0.3 to 0.3, these genes were categorized as ‘diploid’ (0). For other omics data, the somatic mutation and HumanMethylation450 arrays were also acquired from TCGA portal. In this study, the mutation rate and CNA landscape of each patient were evaluated across 33 cancer types. Using Pearson correlation analysis, the relationship between methylation sites and AARS2 expression was assessed.

#### 2.1.3 Immunotherapy cohorts

Two immunotherapy cohorts GSE78220 and GSE100797 were acquired from Gene Expression Omnibus (GEO) platform (https://www.ncbi.nlm.nih.gov/geo/). Both GSE78220 and GSE100797 harbored complete expression and clinical information, melanoma patients with anti‐PD‐1 treatment and adoptive T cell therapy (ACT) treatment, respectively.^13^, ^14^ Based on the RECIST v1.1 standard, the responders and non‐responders were defined as patients with partial response (PR) or complete response (CR) and patients with progressive disease (PD) or stable disease (SD), respectively. Therefore, our study enrolled 15 responders and 13 non‐responders in GSE782208, as well as 8 responders and 13 non‐responders in GSE100797.^15^

### 2.2 Proteomic analysis

With the development of protein quantification, the mass spectrometry (MS)‐derived high‐throughput proteomics is becoming a core technology for large‐scale protein characterization.^16^ The label‐free protein quantification was performed in 40 pairs of tissue from our hospital. In this study, the MaxQuant 1.5.3.17 software was employed to combine and search the MS raw data of each sample, which was retrieved to protein identification and quantitation analysis. Ultimately, the protein expression of potential biomarker molecular AARS2 was available in Table S2. The differences of protein expression were tested by paired‐sample t‐test between tumor and matched adjacent non‐tumor samples. See Supplementary Methods for the detailed depiction.

### 2.3 RNA preparation and quantitative real‐time PCR (qRT‐PCR)

The total RNA was acquired and isolated from liver cells and 20 pairs of tissue using RNAiso Plus reagent (Takara). Then, the quality and expression were quantized via NanoDrop One C. The qRT‐PCR assays were performed by a QuantStudio™ 5 Real‐Time PCR System (Thermo Fisher Scientific Inc.). Using GAPDH as endogenous control to normalize gene expression, which was calculated with ΔCT (Ct mRNA‐Ct GAPDH) approach, and further log2 transformed for analysis. The qRT‐PCR primer sequences were as follows: AARS2 forward: 5′‐AACTTCTGGGAGATGGGGGA‐3′, AARS2 reverse: 5′‐GCTTCCATCTGCCTCTCTGTT‐3′, GAPDH forward: 5′‐GGAGCGAGATCCCTCCAAAAT‐3′, GAPDH reverse: 5′‐GGCTGTTGTCATACTTCTCATGG‐3′.

### 2.4 Western blot (WB) analysis

The liver lysates and cell lysates were collected into EP tubes and then extracted to obtain proteins using RIPA buffer (Solarbio Life Sciences). For WB analysis, total protein was resolved via SDS‐PAGE (10%) in electrophoretic system and further transferred onto nitrocellulose membranes with 0.45 μm. Using 5% non‐fat milk blocked the nitrocellulose membranes for 1 h at indoor temperature. The primary antibodies against AARS2 (1:2000, Cat No. 22696‐1‐AP) and GAPDH (1:1000, Cat No. 5174) were added and incubated these membranes at 4°C overnight. Anti‐AARS2 was purchased from Proteintech Group, and anti‐GAPDH was purchased from Cell Signaling Technology. After washing membranes three times with 1X TBST, the secondary antibody was further used to incubate for 2 h at room temperature. The protein blots were visualized by ECL product (Thermo Fisher Scientific) in dark room after washing membranes three times again.

### 2.5 Tissue immunohistochemistry analysis

Based on our previous study, the immunohistochemistry staining (IHC) and image processing were conducted as standard procedures.^17^ The clinical tissue specimens were fixed with 10% formalin and stored in paraffin embedding once they are in vitro after hepatectomy. The results of IHC analysis were assessed and quantized according to three indicators, including IHC positive area, IHC area density, and IHC score value (H‐Score). The IHC positive area was defined as cumulative optical density IOD value/tissue pixel area and IHC area density was defined as positive area/tissue area.^18^ The H‐Score = (percentage of weak intensity cells) × 1 + (percentage of moderate intensity cells) × 2 + (percentage of strong intensity cells) × 3.^19^ Immunohistochemical scan analysis was conducted by CaseViewer2.4 (3DHISTECH) software for all samples.

### 2.6 Cell culture and small interfering RNA (siRNA) transfection

A total of five HCC cell lines confirmed using short tandem repeat analysis were utilized in this study, including HCC‐LM3, Hep3B, HepG2, Huh7, and SMMC‐7721. These cells were incubated in 5% carbon dioxide (CO_2_) at 37°C and cultured in DMEM medium added with 10% fetal bovine serum (FBS, Gibco) at 37°C. All these cells were employed in qRT‐PCR for screening the optimal one and further performing biology experiments. To accomplish siRNA transfection, three sequences of Si‐AARS2‐1, Si‐AARS2‐2, and Si‐AARS2‐3 were transfected into 30%–40% confluent cells by siRNA‐Mate (GenePharma). The Si‐control and Si‐AARS2 were synthesized by Shanghai GenePharma Co., Ltd. The detailed sequences of these siRNAs are available in Table S3. The transfection efficiency was tested and confirmed using qRT‐PCR and WB analysis.

### 2.7 Cell colony formation and wound healing assay

Cell colony formation and wound healing assays are simple, popular, and highly reproducible approaches to exploring cancer cell proliferation and cell migration in vitro, respectively. To perform cell colony formation, each well was inoculated with 1000 cells in a six‐well plate for 14 days. Subsequently, these cells underwent a series of processes according to instructions, encompassing fixed with 4% paraformaldehyde, stained by 0.1% crystal violet, and visualization. In cell wound healing assay, appropriate HCC cells were inoculated in six‐well plates until reaching 90% confluency after 24 h of transfection. Then cell monolayer was scratched using sterile pipette tips behind serum starvation. The wound closure was photographed by a digital camera under inverted microscope at 0 h and 48 h for later visualization pictures.

### 2.8 The evaluation of oncogenic biological pathway

The Gene Set Variation Analysis (GSVA) is a popular unsupervised approach for assessing variation of gene set enrichment according to gene expression. The classical gene sets of 50 hallmark pathways were obtained from Molecular Signatures Database (MSigDB, https://www.gsea‐msigdb.org/gsea/msigdb/). Using GSVA method, the activity of oncogenic pathway was estimated and quantized. The correlation analysis displayed the relationship between AARS2 and oncogenic pathways. In addition, the top 500 significant correlation genes with AARS2 were filtered and then employed to GO and KEGG enrichment analyses. The gene sets were also derived from MSigDB resource (kegg.v7.5.1 and go.v7.5.1). Gene sets with FDR <0.05 were considered to be significantly active biological pathways.

### 2.9 The estimation of immune microenvironment and drug response

According to Charoentong et al.^20^ research, a total of 28 immune cell types were enrolled to reflect the immune microenvironment status. Using single sample gene set enrichment analysis (ssGSEA) method depicted the relative infiltration abundance of immune cells. The correlation between AARS2 expression and immune cell activity was estimated by the Pearson correlation coefficient. To seek more ways for cancer patients, 138 anticancer drugs were retrieved from previous studies.^21^ The half‐maximum inhibitory concentration (IC50) value was served as predictive indicator to evaluate drug sensitivity. For each TCGA sample, the imputed drug response was calculated and quantized. Then, the correlation between predicted drug response and AARS2 expression was assessed using the Pearson correlation coefficient. The candidate drugs were taken into harboring potential targeting for AARS2 based on the top 10 | r | and FDR <0.05.

### 2.10 Statistical analysis

For two continuous variables, the relationship was estimated by Pearson correlation analysis. Correlation value with | r | >0.3 and FDR <0.05 was considered as significance in multi‐omics analysis part. The optimal cut‐off point was determined by *survminer* package, and patients were further stratified into high‐ and low‐groups. The *survival* package was utilized to perform Kaplan–Meier analysis, and the significance was tested via log‐rank test. The receiver operating characteristic (ROC) curves were depicted using *pROC* package, and the area under ROC value (AUC) was employed to assess the accuracy of immunotherapy response. For non‐paired samples, the differential expression of AARS2 was compared using the Wilcoxon rank sum test or two‐tailed unpaired Student's *t*‐test. For paired samples, paired‐sample *t*‐test was used to compare the differences of AARS2 expression between the two groups. The adjusted *p* value was calculated by the Benjamini–Hochberg multiple test correction and defined as FDR value. All *p* values were two‐tailed, and the *p* < 0.05 indicated statistical significance. All graphs, data processing, and statistical analysis were performed by R software (Version 4.1.2) and GraphPad Prism 8.0.1 software (GraphPad Software).

## 3 RESULTS

### 3.1 The genomics landscape of AARS2 in pan‐cancer

**FIGURE 1:**
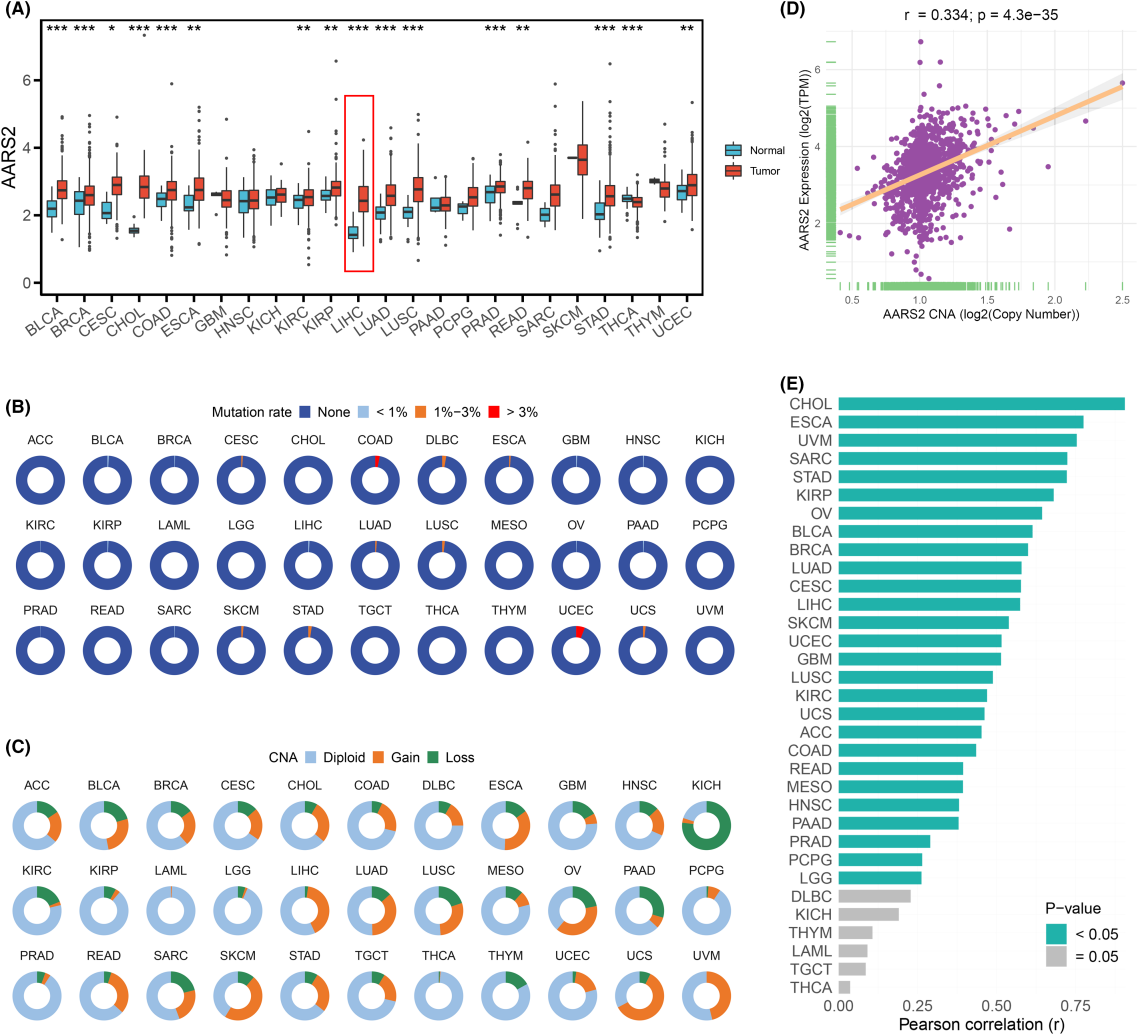
The genomics landscape of AARS2 in human cancers. (A) The expression of AARS2 between normal and tumor tissues in pan‐cancer. (B) The mutation rate of AARS2 across 33 cancer types. (C) The copy number alternation frequency of AARS2 across 33 cancer types. (D) The correlation between copy number alternation and AARS2 expression in tumor cells. (E) The correlation between copy number alternation and AARS2 expression in pan‐cancer. *p < 0.05; **p < 0.01; ***p < 0.001.

Based on gene expression level, AARS2 displayed frequently elevated mRNA expression across most cancers, especially in HCC types (Figure 1A). To further decode the factors impacting AARS2 expression, the genomic landscape of AARS2 was explored across cancers. The mutation of AARS2 was known as harboring links with leukodystrophy and cardiomyopathy diseases, while our findings suggested it was likely to have no significant association with cancers owing to low mutation rate (Figure 1B). Apart from mutation, gene methylation is another potential key indicator that regulates molecular expression and triggers carcinogenesis. A total of 15 AARS2 methylation sites were existed in HumanMethylation450 array. Correlation analysis elucidated that AARS2 expression was negatively associated with DNA methylation across multiple cancers (Figure S1A). As only partial methylation sites harbored significant correlation, CNA of AARS2 was further investigated across pan‐cancer. Notably, the CNA of AARS2 occurred significant gain and loss in multiple cancers, such as KICH, SKCM, and UVM (Figure 1C). Using tumor cell information, a novel insight was decoded that AARS2 expression presented a positive correlation with CNA (Figure 1D). Meanwhile, similar findings were also elaborated that AARS2 expression is positively associated with CNA in most cancers, including CHOL and LIHC (Figure 1E). Therefore, the upregulation of AARS2 might be extensively driven by CNA rather than gene mutation or DNA methylation.

### 3.2 Frequent high expression of AARS2 associated with dismal prognosis in HCC

**FIGURE 2:**
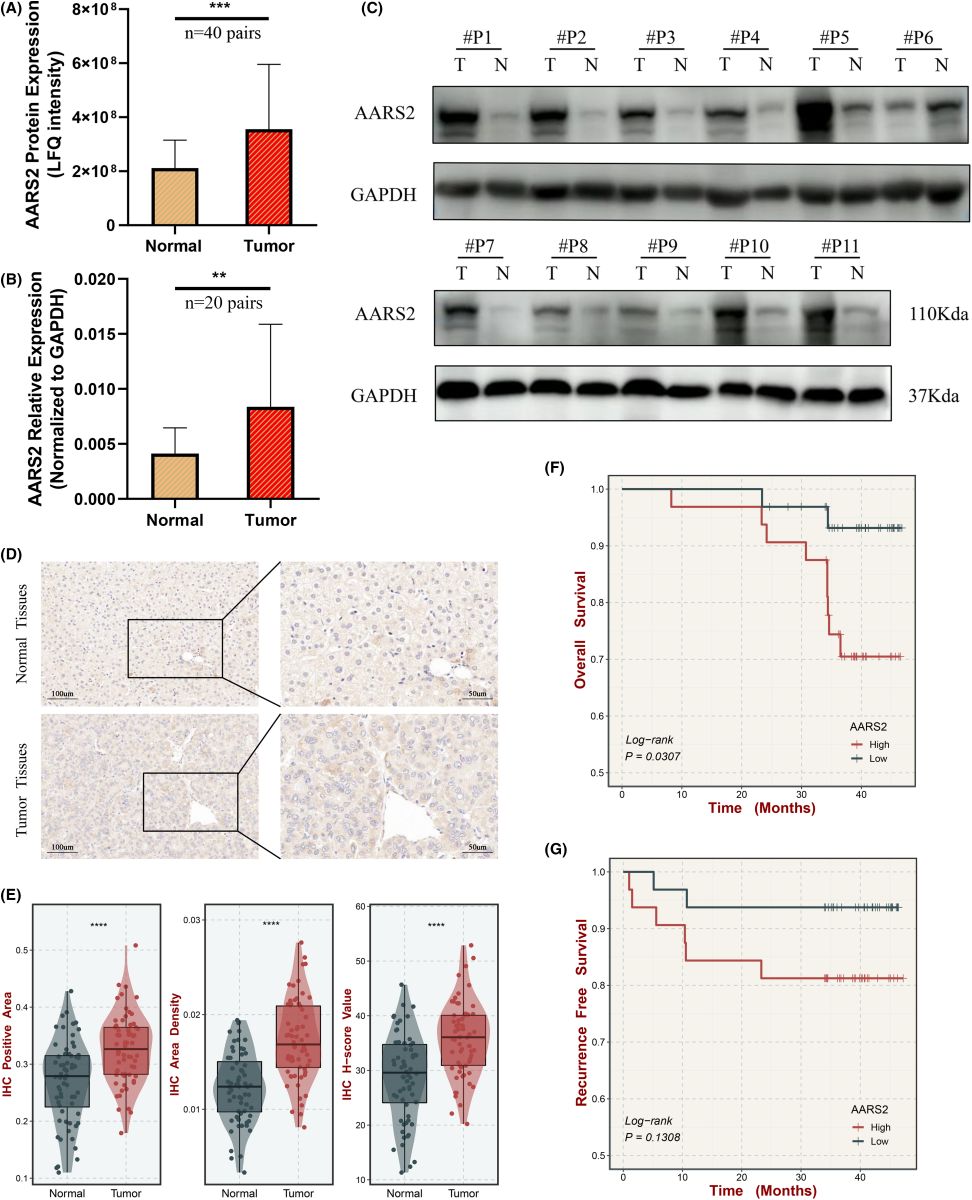
The AARS2 is elevated expressed in human HCC tissues. (A) The difference of AARS2 protein expression in 40 tumor and matched adjacent non‐tumor samples from HCC patients. (B) The difference of AARS2 mRNA expression in 20 paired samples. (C) The expression levels of AARS2 in 11 paired tissues from HCC patients detected by western blotting. (D) Representative immunohistochemical staining of AARS2 in 64 paired tissues from HCC patients. Scale bar is 100 μm and 50 μm, respectively. (E) The distribution of IHC positive area, IHC area density, and IHC score value in 64 paired tissues. (F, G) Kaplan–Meier survival curve of AARS2 for overall survival (F) and recurrence free survival (G).

Owing to AARS2 displayed the most significant difference in HCC, further validation and exploration of its potential biological value were performed. The proteomics analysis indicated that the protein level of AARS2 was predominantly upregulated in histopathological HCC tissues (Figure 2A). Subsequently, we performed qRT‐PCR and WB to validate the elevated expression of AARS2 at mRNA and protein levels. The results of qRT‐PCR analysis demonstrated that AARS2 was conspicuously upregulated in 20 paired HCC samples (Figure 2B). Consistent with proteomic conclusion, WB analysis proved AARS2 had pronouncedly high expression again in HCC tumor samples (Figure 2C). Moreover, the IHC analysis also exhibited high expression of AARS2 in tumors relative to normal tissues (Figure 2D). Three indicators—IHC positive area, IHC area density, and H‐Score—all supported AARS2 expression dramatically increased in 64 paired tumor tissues (Figure 2E). These results indicated that AARS2 may perform oncogenic functions in HCC. To better understand the clinical implications of AARS2 in HCC, patients were divided into high‐ and low‐expression groups according to the optimal cut‐off value. The Kaplan–Meier survival curves indicated patients with high AARS2 expression presented the trend of poor clinical outcomes at overall survival (OS) and recurrence‐free survival (RFS) (Figure 2F,G). Taken together, AARS2 might serve as a novel oncogenic gene and perform a tumor‐accelerative role in HCC.

### 3.3 The exploration of biological function of AARS2

**FIGURE 3:**
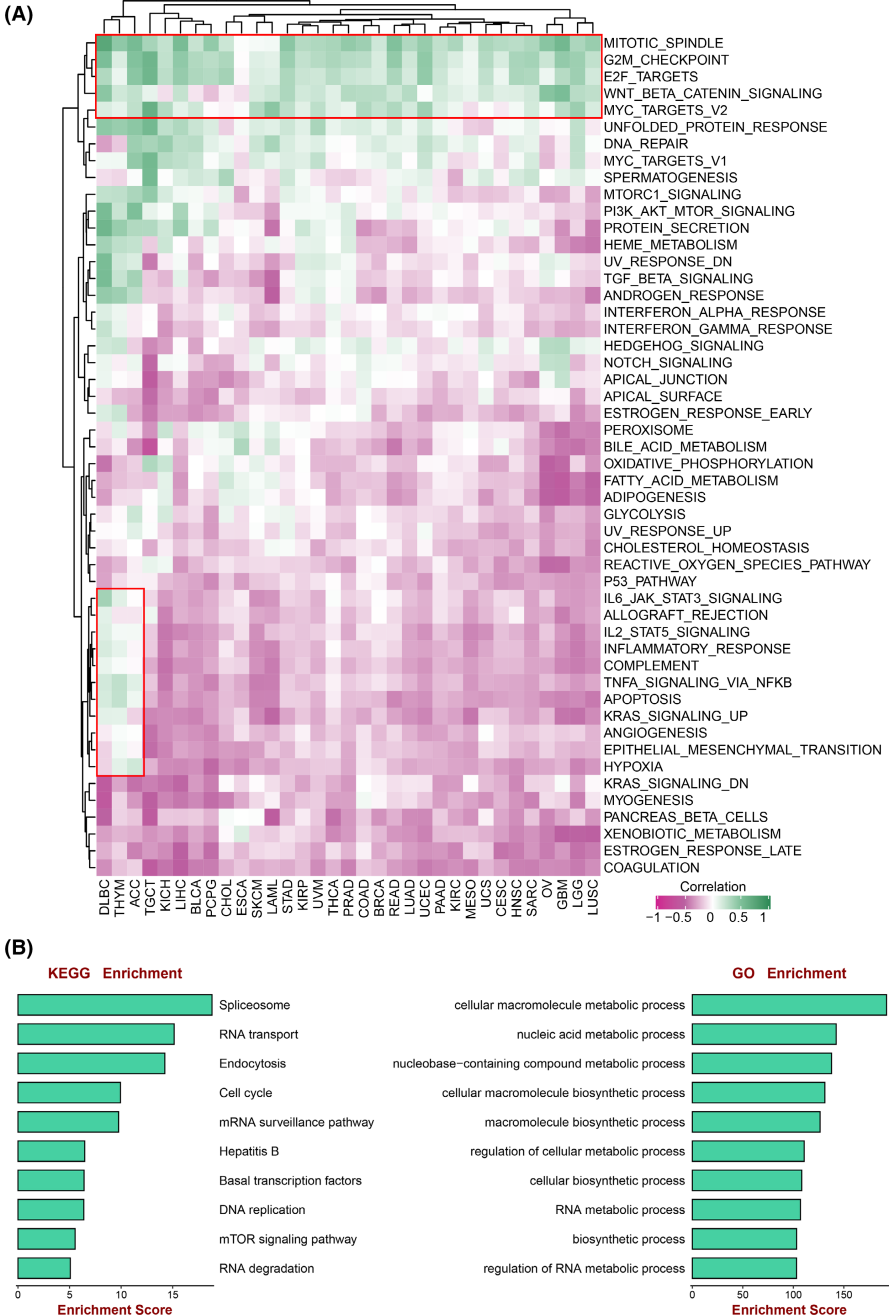
The biological characterization of AARS2 in cancer activity. (A) The relationship between AARS2 expression and 50 cancer associated pathways activity across 33 cancer types. (B) GO and KEGG enrichment analysis to explore the biological characterization of AARS2.

To further shed light on the biological function, the relationship between AARS2 and oncogenic pathway activity was calculated and assessed. As illustrated in Figure 3A, AARS2 was tightly associated with multiple oncogenic pathways across most of cancers. Interestingly, AARS2 was likely to perform multiple functional roles in different cancers. The results indicated that AARS2 was predominantly positively correlated with immune inflammatory pathways, such as IL6 JAK STAT3 Signaling in DLBC, TNFα signaling via NF‐κB in THYM, DLBC, and ACC. However, AARS2 was negatively related to immune‐inflammatory pathways in other cancers (Figure 3A). In addition, AARS2 also displayed the consensus and same biological activity across almost all cancers, which harbored conspicuous positive relationship with proliferation‐related pathways such as G2M checkpoint, Wnt/β‐catenin pathway, and MYC Targets V2 (Figure 3A). Furthermore, GO and KEGG enrichment analyses suggested that AARS2 was closely links with cell cycle, mTOR signaling pathway, and cancer metabolism activity (Figure 3B). All the above results indicated that AARS2 was deemed to perform a crucial role in tumor progression.

### 3.4 AARS2 promotes cell proliferation and migration of HCC cells

**FIGURE 4:**
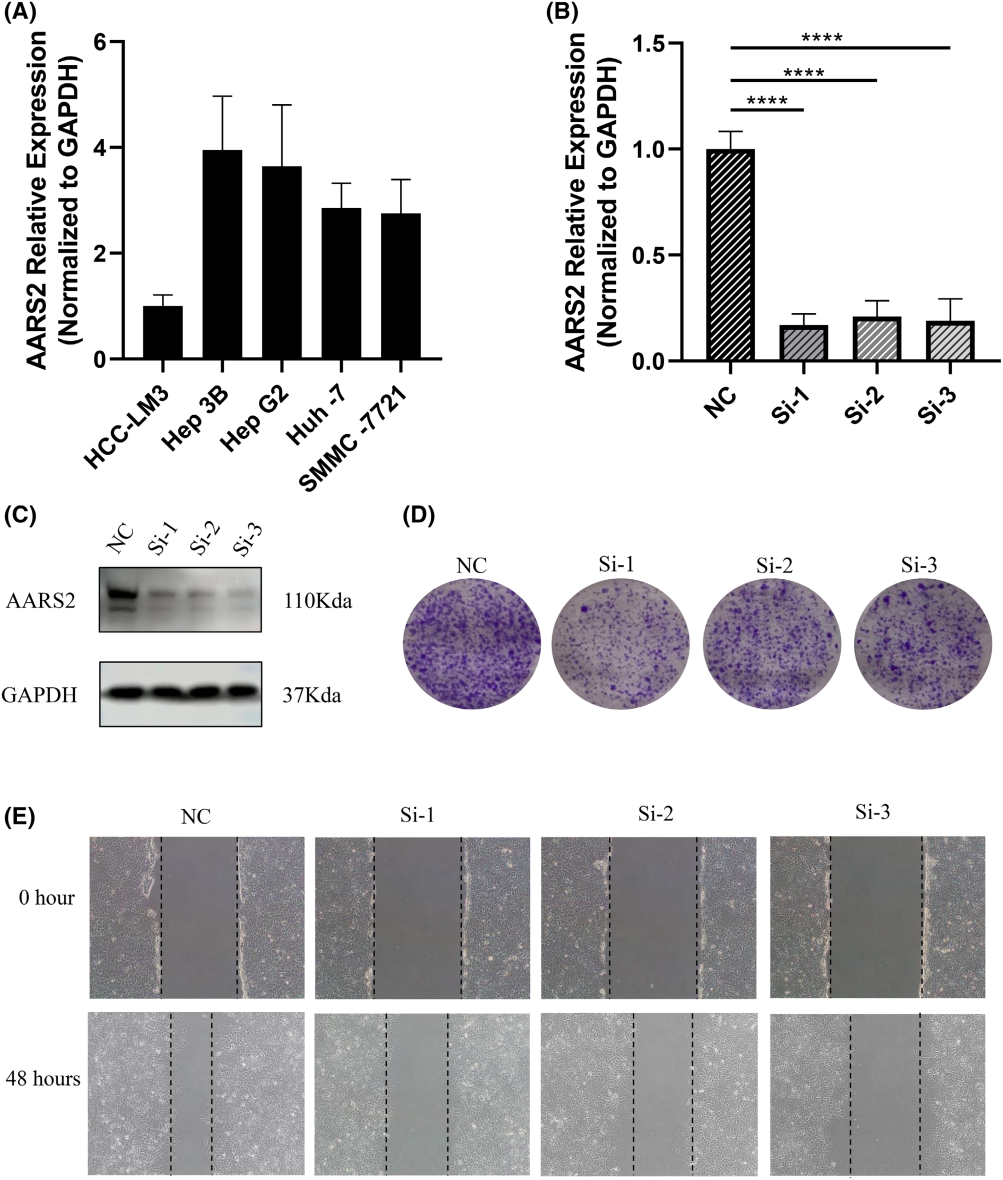
The deficiency of AARS2 inhibited cell proliferation and cell migration in HCC. (A) The expression of AARS2 in five liver cell lines at mRNA level. (B) The qRT‐PCR for testing the expression of AARS2 in Hep 3B cells with siRNA transfection. (C) The western blot for testing the protein expression of AARS2 in Hep 3B cells with siRNA transfection. (D) Cell colony formation for assessing cancer cell proliferation. (E) The wound healing assay for evaluating cell migration.

The role of AARS2 was further investigated in HCC cells to elaborate on its biological function. Firstly, the expression of AARS2 was detected in five distinct HCC cell lines using qRT‐PCR analysis. The Hep 3B cell harbored the highest basal expression of AARS2 and was then enrolled in subsequent experimental studies (Figure 4A). Next, Hep 3B cells were treated with siRNAs, and AARS2 expression was suppressed, which was confirmed by qRT‐PCR analysis (Figure 4B). The WB analysis validated the efficacy of siRNA transfection again and proved low expression of AARS2 at protein level (Figure 4C). The cell colony formation was widely used to estimate cell proliferation, and the wound healing assay was an effective approach to reflecting the ability of cell migration. Based on cell colony formation and wound healing assay, the results indicated that AARS2 suppression markedly decreased cell proliferation and migration (Figure 4D,E). Therefore, AARS2 deficiency inhibited the tumorigenicity of human HCC cells and further supported its oncogenic gene role.

### 3.5 Clinical implications of AARS2 for immunotherapy

**FIGURE 5:**
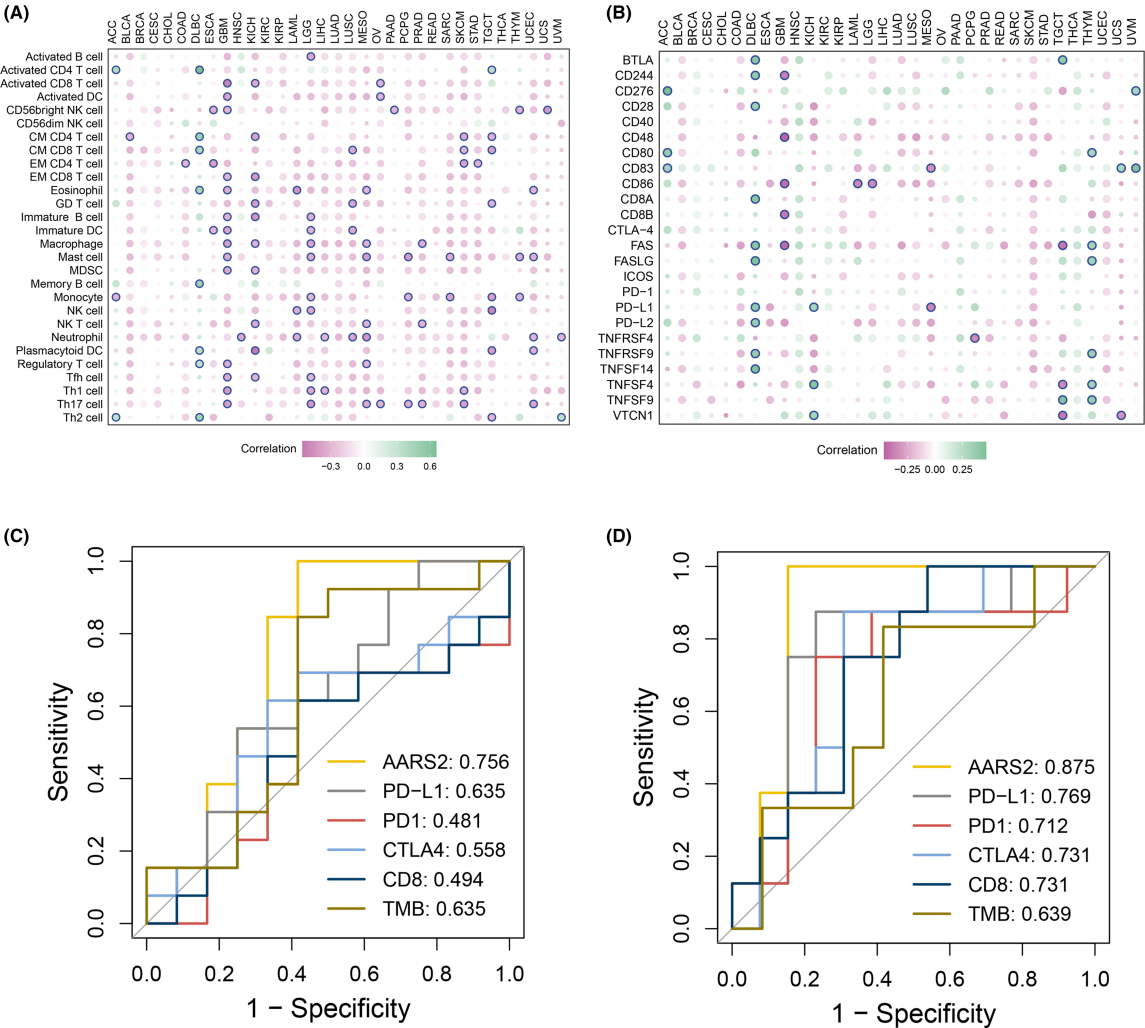
The assessment of AARS2 for immunotherapy indicator. (A) Correlation between AARS2 expression and 28 immune cells infiltration in pan‐cancer. (B) Correlation between AARS2 expression and 24 immune checkpoints expression in pan‐cancer. (C) The assessment of accuracy in predicting immunotherapy efficacy compared AARS2 with classical biomarkers in GSE782208. (D) The assessment of accuracy in predicting immunotherapy efficacy compared AARS2 with classical biomarkers in GSE100797.

After decoding genomic characteristics and exploring the biological function of AARS2, whether it can serve as a biomarker to promote clinical transformation should be further concerned. As we known, immune microenvironment performed a core role in tumor immunotherapy.^22^ Meanwhile, AARS2 is enriched in distinct immune‐related pathways across multiple cancers. Our study evaluated the relationship between AARS2 and 28 immune cells and 24 immune checkpoints, respectively. The AARS2 presented a significant correlation with immune cell infiltrations, especially in DLBC, GBM, KICH, and SKCM (Figure 5A). Similar findings were also observed that AARS2 displayed tight correlation with immune checkpoints in some cancers, such as DLBC, GBM, and SKCM (Figure 5B). Therefore, it is worthy to presuming AARS2 might be a potential biomarker for assessing immunotherapy response. Subsequently, we enrolled two independent immunotherapy cohorts and some classical biomarkers for predicting immunotherapy response, including CD8, PD‐1, PD‐L1, CTLA4, and TMB. The ROC curves demonstrated that AARS2 displayed the highest accuracy in assessing immunotherapy efficacy compared with these classical biomarkers (Figure 5C,D). All these results elaborated that AARS2 could serve as a promising biomarker for assessing immunotherapy efficacy, especially in SKCM.

### 3.6 The value of prognosis and drugs treatment across pan‐cancer

**FIGURE 6:**
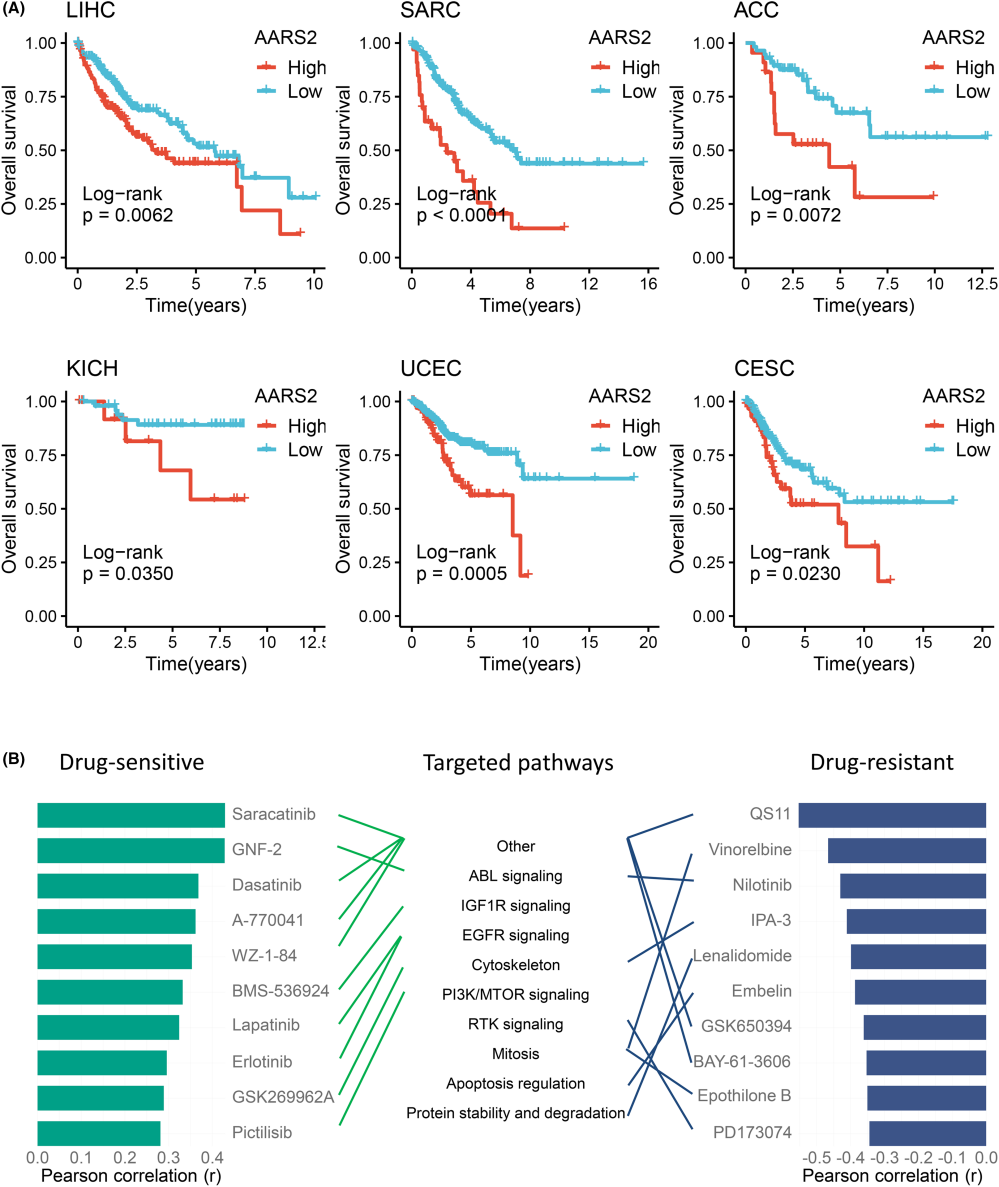
The prognosis value and drug development according to AARS2. (A) The prognosis implication of AARS2 in multiple cancers, such as LIHC, SARC, ACC, KICH, UCEC, and CESC. (B) From the perspective of AARS2 expression, the identification of potential drugs and targeted pathway for cancers.

To future decipher the clinical value of AARS2, our study performed survival analysis in multiple cancers. The AARS2 displayed essential prognosis implication and supported the above conclusion elevated expression with poor clinical outcome again, especially in LIHC, SARC, ACC, KICH, UCEC, and CESC (Figure 6A). From the perspective of AARS2 expression, our study made efforts on identifying potential drugs and targeted pathway for cancers. According to the correlation between AARS2 expression and the IC50 value of drugs, novel insights were proposed for cancer drug therapy. Obviously, the AARS2 might promote drug sensitivity of Saracatinib, GNF‐2, Dasatinib, A‐770041, WZ‐1‐84, BMS‐536924, Lapatinib, Erlotinib, GSK269962A, and Pictilisib and enhance drug resistance of QS11, Vinorelbine, Nilotinib, IPA‐3, Lenalidomide, Embelin, GSK650394, BAY‐61‐3606, Epothilone B, and PD173074 (Figure 6B). Meanwhile, an interesting finding was that AARS2 may perform distinct drug responses to the common targeted pathway. The ABL signaling was characterized as targeted pathway of GNF‐2 and Nilotinib, whereas AARS2 enhanced the drug sensitivity of GNF‐2 while decreased the drug sensitivity of Nilotinib (Figure 6B). Overall, these novel findings might stimulate drug development and facilitate clinical efficacy in human cancers.

## 4 DISCUSSION

There is a significant increase in focus within pan‐cancer research to identify potential biomarkers.^23^, ^24^ Comprehensive explorations of novel molecules could guide deeper understanding of cancer and facilitate targeted therapy.^25^ The ARSs have been reported harboring crucial role in human cancer, while AARS2 as one of them remains almost a blank space in human cancer.^8^, ^26^ In this study, the AARS2 gene was broadly investigated regarding genomic alterations, biological function, prognosis, and therapeutic implications across 33 human cancer types.

AARS2 is one of the mitochondrial enzymes encoded by the nuclear gene, which is responsible for the transferring tRNA‐ala with alanine and further impacts protein synthesis. Mutations in AARS2 have been confirmed as a key factor underlying cardiomyopathies and leukoencephalopathies.^11^, ^27^ Recently, an oncology study has proposed that AARS2 could serve as a prognostic indicator for predicting survival in colon cancer.^12^ To decipher the potential value of AARS2 in human cancers, our study initially determined AARS2 is elevated in tumor tissues compared to normal tissues at both mRNA and protein levels, especially in HCC. Genomic alterations are usually associated with aberrant gene expression, which might lead to the changes of cell behavior and drive carcinogenesis.^28^ Subsequently, the genomic landscape of AARS2 was depicted in 33 human cancer types, including mutations, CNA, and gene methylation. The mutations of AARS2 were rather infrequent, which might be links with the appearance of fatal diseases once its variation.^29^ Meanwhile, compared to methylation, the CNA variations of AARS2 were conspicuous, and the correlations between CNA and gene expression were also strong. Therefore, a novel insight was provided that the upregulation of AARS2 was mainly driven by CNA rather than mutation and methylation.

According to biological function analysis, the abnormal expression of AARS2 was prominently associated with activity of oncogenic pathways. The AARS2 displayed consensus biological activity and was widely involved in proliferation‐related activity across almost of all cancers. Interestingly, AARS2 also appeared poised to carry out diverse functional roles in different cancers. For example, AARS2 was primarily implicated in immune inflammatory pathways in THYM and DLBC. To observe the detailed biological behavior in specific cancer, cell colony formation and wound healing assays were conducted in Hep 3B. The knockdown of AARS2 markedly inhibited cell proliferation and cell migration, which was also the first report. Overall, the AARS2 was an oncogenic gene in HCC and might perform a similar role in most cancers.

In addition, HCC patients with high AARS2 expression harbored poor clinical outcome according to IHC staining and survival curves. Elevated expression of AARS indicated dismal prognosis in multiple cancers, such as SARC, ACC, KICH, UCEC, and CESC. Given its role as an oncogene, whether AARS2 could serve as a promising biomarker to promote clinical transformation should be concerned. The immune microenvironment fulfills essential functions in regulating immune interactions and affecting immunotherapy efficacy.^30^ After assessing the immune microenvironment, the AARS2 also performed tight association with immune infiltration and immune checkpoint expression. In two immunotherapy cohorts, AARS2 displayed more accuracy at predicting immunotherapy response relative to classical biomarkers, such as CD8, PD‐1, PD‐L1, CTLA4, and TMB. All these novel findings indicated that AARS2 might be a promising biomarker for predicting prognosis and immunotherapy responses in cancer. Moreover, our study provided that the AARS2 might promote the drug sensitivity of Saracatinib, GNF‐2, Dasatinib, A‐770041, WZ‐1‐84, BMS‐536924, Lapatinib, Erlotinib, GSK269962A, and Pictilisib while strengthening resistance to QS11, Vinorelbine, Nilotinib, IPA‐3, Lenalidomide, Embelin, GSK650394, BAY‐61‐3606, Epothilone B, and PD173074. For instance, the small‐molecule inhibitor of ABL named GNF‐2 overcomes off‐target toxicities in solid malignancies, improving clinical efficacy.^31^ Combining anti‐PD‐L1 drug and nilotinib, a tyrosine kinase inhibitor targeting BCR‐ABL, markedly increases survival of leukemic mice.^32^ These potential drugs and targeted pathways brought a new direction for cancer therapy.

Our study provided a well‐rounded characterization of AARS2, including genomic characterizations, prognosis, biological function, immunotherapy response, and drug treatment associations. The AARS2 was attractive for improving prognosis and facilitating clinical management, but some limitations should be clarified. Firstly, all the samples enrolled in this research were retrospective, and some prospective studies should be applied to validate new findings. Secondly, a multicenter and large‐sample dataset containing eligible patients with immunotherapy needs to be further employed to assess the clinical efficacy. Thirdly, more in vivo experiments should be conducted, especially the animal experiments and clinical trial research.

Taken together, the overexpression of AARS2 might be mainly caused by genomic CNA and is prone to unfavorable prognosis as well as proliferation phenotype. The deficiency of AARS2 conspicuously inhibited cell proliferation and cell migration in HCC. The AARS2 was revealed to be a novel and promising biomarker for prognosis and immunotherapy responses in human cancers. This study increased the understanding of AARS2 and provided the potential target of timely intervention across cancers.

## AUTHOR CONTRIBUTIONS

**Long Liu:** Conceptualization (equal); data curation (equal); formal analysis (equal); investigation (equal); methodology (equal); software (equal); validation (equal); visualization (equal); writing – original draft (equal). **Jie Gao:** Project administration (equal); supervision (equal); writing – review and editing (equal). **Xudong Liu:** Conceptualization (equal); project administration (equal); supervision (equal); writing – review and editing (equal). **Feng Zhang:** Writing – review and editing (equal). **Bowen Hu:** Writing – review and editing (equal). **Huapeng Zhang:** Funding acquisition (equal); writing – review and editing (equal). **Zhihui Wang:** Writing – review and editing (equal). **Hongwei Tangi:** Writing – review and editing (equal). **Ji Hua Shi:** Writing – review and editing (equal). **Shuijun Zhang:** Conceptualization (equal); data curation (equal); funding acquisition (equal); investigation (equal); methodology (equal); project administration (equal); resources (equal); software (equal); supervision (equal); validation (equal); visualization (equal); writing – original draft (equal).

## FUNDING INFORMATION

This study was supported by the National Natural Science Foundation of China (82170670), Youth Project of National Natural Science Foundation of China (82103282), Youth Project of National Natural Science Foundation of China (82103211), Henan Charity General Federation of Hepatobiliary Care Fund (GDXZ2023002), Youth Project of Henan Province Medical Science and Technology Research (SBGJ202103061), Henan Province Medical Science and Technology Research Plan (LHGJ20200357).

## CONFLICT OF INTEREST STATEMENT

The authors declare that they have no competing interests.

## CONSENT FOR PUBLICATION

We have obtained consents to publish this paper from all the participants of this study.

## Supporting information


Data S1

Figure S1

Table S1

Table S2

Table S3

## Data Availability

Public data used in this work can be acquired from The Cancer Genome Atlas (TCGA, https://portal.gdc.cancer.gov/); Gene Expression Omnibus (GEO, https://www.ncbi.nlm.nih.gov/geo/); Molecular Signatures Database (MSigDB, https://www.gsea‐msigdb.org/gsea/msigdb/index.jsp); and Cancer Cell Line Encyclopedia (CCLE, https://portals.broadinstitute.org/ccle/).
