# Supplementary material for: AARS2 as a novel biomarker for prognosis and its molecular characterization in pan‐cancer
Source: Cancer Med. 2023 Nov 21;12(23):21531–44. doi: 10.1002/cam4.6682 (PMC10726843; doi:10.1002/cam4.6682)
Supplement: Supplementary file 1 — Data S1 [file CAM4-12-21531-s002.docx]

**Supplementary Methods**

**The** **abbreviation of 33 cancer types in TCGA**

In this study, 33 different TCGA cohorts were enrolled, each cohort represents one specific cancer type. All results from multi-cancers were based on these data (<http://cancergenome.nih.gov/>). The detailed abbreviation of 33 cancer types appeared in our works was as following: adrenocortical carcinoma (ACC); bladder urothelial carcinoma (BLCA); breast cancer (BRCA); cervical squamous cell carcinoma and endocervical adenocarcinoma (CESC); cholangiocarcinoma (CHOL); colon adenocarcinoma (COAD); lymphoid neoplasm diffuse large b-cell lymphoma (DLBC); esophageal carcinoma (ESCA); glioblastoma multiforme (GBM); head and neck squamous carcinoma (HNSC); kidney chromophobe (KICH); kidney renal clear cell carcinoma (KIRC); kidney renal papillary cell carcinoma (KIRP); acute myeloid leukemia (LAML); brain lower grade glioma (LGG); liver hepatocellular carcinoma (LIHC); lung adenocarcinoma (LUAD); lung squamous cell carcinoma (LUSC); mesothelioma (MESO); ovarian serous cystadenocarcinoma (OV); pancreatic adenocarcinoma (PAAD); pheochromocytoma and paraganglioma (PCPG); prostate adenocarcinoma (PRAD); rectum adenocarcinoma (READ); sarcoma (SARC); skin cutaneous melanoma (SKCM); stomach adenocarcinoma (STAD); testicular germ cell tumors (TGCT); thyroid carcinoma (THCA); thymoma (THYM); uterine corpus endometrial carcinoma (UCEC); uterine carcinosarcoma (UCS); uveal melanoma (UVM).

**The inclusion criteria and usage of specimens**

A total of 71 pairs of fresh tissue and 64 pairs of paraffin-embedded specimens were collected from patients in The First Affiliated Hospital of Zhengzhou University. Among of these specimens, 20 pairs for Quantitative real-time PCR (qRT-PCR), 11 pairs for western blot (WB) analysis, 40 pairs proteomic analysis, and 64 pairs for immunohistochemistry (IHC). The inclusion and exclusion criteria were as following: (1) no preoperative radiotherapy and chemotherapy; (2) primary HCC; (3) no other types of tumors. All specimens collected during surgery operation were immediately snap frozen in liquid nitrogen and then stored at -80°C. The overall survival (OS) and recurrence free survival (RFS) were served as endpoint events in the process of follow-up. The RFS was deemed as the time from tissue acquisition to date of recurrence, and the OS was deemed as the time from tissue acquisition to date of death. The clinical information of each patient was stored in Table S1.

**Proteomic analysis**

With the development of protein quantification technology, the mass spectrometry (MS)-based high-throughput proteomics is becoming a core technique for large-scale protein characterization. Using label free quantitative mass spectrometry, tissue proteins were obtained by SDT [4% (w/v) SDS, 0.1M DTT, and 100mM Tris/HCl], further quantified by BCA approach. The Filter Aided Proteome Preparation (FASP) approach was used to trypsin enzymolysis and C18 Cartridge was employed to desalinate the peptide. Subsequently, peptide after lyophilization was redissolved with 40 μL 0.1% formic acid and quantified based on OD280. Single sample was divided into 15 fractions via RP-HPLC and combined into 5 fractions by orthogonal combination approach. LC-MS/MS analysis was conducted on Q Exactive mass spectrometer (QE-HF-X, Thermo Scientific), which was coupled to Nano-LC (Easy1200, Thermo Fisher Scientific). According to protocol, the peptides were loaded onto a reverse phase trap column, connected to the C18-reversed phase analytical column in buffer A, and separated with a linear gradient of buffer B by flow rate (300 nL/min) performed byIntelliFlow technology. Using a data-dependent top10 approach to generate MS data based on choose the most abundant precursor ions from the survey scan for HCD fragmentation. Ultimately, the MaxQuant 1.5.3.17 software was employed to combine and search the MS raw data of each sample, which was retrieved to protein identification and quantitation analysis.
