# Supplementary material for: AARS2 as a novel biomarker for prognosis and its molecular characterization in pan‐cancer
Source: Cancer Med. 2023 Nov 21;12(23):21531–44. doi: 10.1002/cam4.6682 (PMC10726843; doi:10.1002/cam4.6682)
Supplement: Supplementary file 2 — Figure S1 [file CAM4-12-21531-s001.docx]

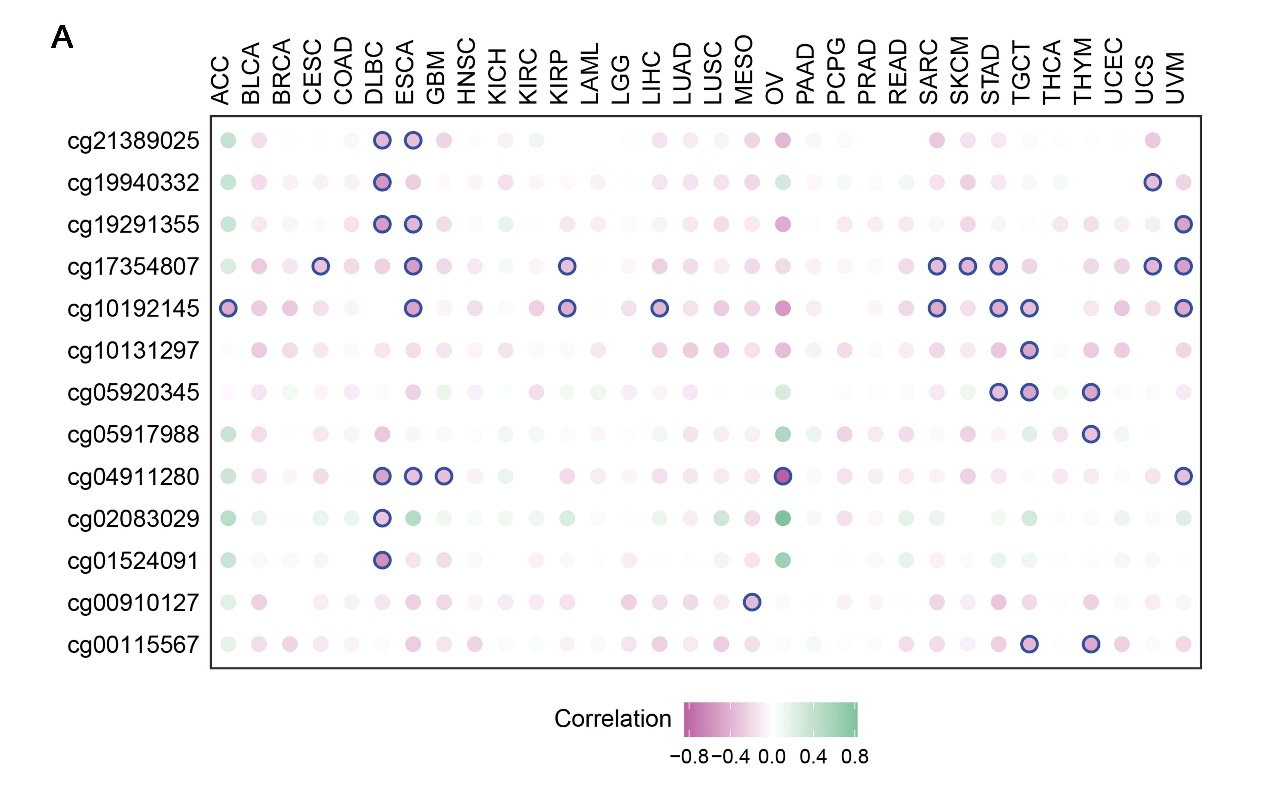


**Figure S1. The methylation correlation of AARS2 across pan-cancer.** **(A).** Pearson correlation between the expression of AARS2 and methylation beta value of 15 corresponding CpG sites.
