# Supplementary material for: AARS2 as a novel biomarker for prognosis and its molecular characterization in pan‐cancer
Source: Cancer Med. 2023 Nov 21;12(23):21531–44. doi: 10.1002/cam4.6682 (PMC10726843; doi:10.1002/cam4.6682)
Supplement: Supplementary file 3 — Table S1 [file CAM4-12-21531-s003.docx]

| **Table S1. The details of clinical information in our HCC cohort.** | |
| --- | --- |
| **Total (%)** | 64 (100.0%) |
| **Age (year)** |  |
| <60 | 48 (75.0%) |
| >=60 | 16 (25.0%) |
| **Gender** |  |
| Male | 52 (81.2%) |
| Female | 12 (18.8%) |
| **Alpha fetal protein level (ng/ml)** |  |
| <20 | 25 (39.1%) |
| >=20 | 37 (57.8%) |
| NA | 2 (3.1%) |
| **Greatest tumor diameter (cm)** |  |
| <5 | 36 (56.3%) |
| >=5 | 26 (40.6%) |
| NA | 2 (3.1%) |
| **Total tumor diameter (cm)** |  |
| <5 | 35 (54.7%) |
| >=5 | 34 (45.3%) |
| **Microvascular invasion** |  |
| Yes | 14 (21.9%) |
| No | 49 (76.5%) |
| NA | 1 (1.6%) |
| **Recurrence free status** |  |
| Yes | 8 (12.5%) |
| No | 56 (87.5%) |
| **Survival status** |  |
| Alive | 53 (82.8%) |
| Dead | 11 (17.2%) |
