# Supplementary material for: AARS2 as a novel biomarker for prognosis and its molecular characterization in pan‐cancer
Source: Cancer Med. 2023 Nov 21;12(23):21531–44. doi: 10.1002/cam4.6682 (PMC10726843; doi:10.1002/cam4.6682)
Supplement: Supplementary file 4 — Table S2 [file CAM4-12-21531-s004.docx]

| **Table S2. The AARS2 protein expression in our proteomic cohort.** | | | |
| --- | --- | --- | --- |
| Normal Tissues | Protein Expression | Tumor Tissues | Protein Expression |
| LFQ intensity B1-01 | 185410234 | LFQ intensity A1-01 | 715125607 |
| LFQ intensity B1-02 | 149916515 | LFQ intensity A1-02 | 712196242 |
| LFQ intensity B1-03 | 82835545 | LFQ intensity A1-03 | 291465973 |
| LFQ intensity B1-04 | 242398618 | LFQ intensity A1-04 | 259664164 |
| LFQ intensity B1-05 | 93095152 | LFQ intensity A1-05 | 316155342 |
| LFQ intensity B1-06 | 38517252 | LFQ intensity A1-06 | 529915639 |
| LFQ intensity B1-07 | 182589031 | LFQ intensity A1-07 | 24158199 |
| LFQ intensity B1-08 | 194042153 | LFQ intensity A1-08 | 93734479 |
| LFQ intensity B1-09 | 71470789 | LFQ intensity A1-09 | 127077435 |
| LFQ intensity B1-10 | 217615450 | LFQ intensity A1-10 | 49758242 |
| LFQ intensity B1-11 | 98511039 | LFQ intensity A1-11 | 390586882 |
| LFQ intensity B1-12 | 230221109 | LFQ intensity A1-12 | 120744772 |
| LFQ intensity B1-13 | 418493726 | LFQ intensity A1-13 | 658998219 |
| LFQ intensity B1-14 | 254893233 | LFQ intensity A1-14 | 633906106 |
| LFQ intensity B1-15 | 261911244 | LFQ intensity A1-15 | 146211442 |
| LFQ intensity B1-16 | 228328138 | LFQ intensity A1-16 | 458681435 |
| LFQ intensity B1-17 | 192921541 | LFQ intensity A1-17 | 659817451 |
| LFQ intensity B1-18 | 211683404 | LFQ intensity A1-18 | 367857689 |
| LFQ intensity B1-19 | 225658513 | LFQ intensity A1-19 | 260454813 |
| LFQ intensity B1-20 | 398893012 | LFQ intensity A1-20 | 353026576 |
| LFQ intensity B2-01 | 208325808 | LFQ intensity A2-01 | 90124801 |
| LFQ intensity B2-02 | 171928769 | LFQ intensity A2-02 | 563645362 |
| LFQ intensity B2-03 | 72439506 | LFQ intensity A2-03 | 370116486 |
| LFQ intensity B2-04 | 173988897 | LFQ intensity A2-04 | 252117576 |
| LFQ intensity B2-05 | 201301200 | LFQ intensity A2-05 | 201090928 |
| LFQ intensity B2-06 | 136642247 | LFQ intensity A2-06 | 122216262 |
| LFQ intensity B2-07 | 71572716 | LFQ intensity A2-07 | 184003015 |
| LFQ intensity B2-08 | 154918940 | LFQ intensity A2-08 | 262922384 |
| LFQ intensity B2-09 | 210453930 | LFQ intensity A2-09 | 237049853 |
| LFQ intensity B2-10 | 172103900 | LFQ intensity A2-10 | 740338538 |
| LFQ intensity B2-11 | 438329680 | LFQ intensity A2-11 | 952528547 |
| LFQ intensity B2-12 | 264327232 | LFQ intensity A2-12 | 349035136 |
| LFQ intensity B2-13 | 420365994 | LFQ intensity A2-13 | 385830201 |
| LFQ intensity B2-14 | 340709779 | LFQ intensity A2-14 | 93402498 |
| LFQ intensity B2-15 | 117467849 | LFQ intensity A2-15 | 134406865 |
| LFQ intensity B2-16 | 259428819 | LFQ intensity A2-16 | 773201967 |
| LFQ intensity B2-17 | 231768982 | LFQ intensity A2-17 | 480810306 |
| LFQ intensity B2-18 | 177867850 | LFQ intensity A2-18 | 583406068 |
| LFQ intensity B2-19 | 442226207 | LFQ intensity A2-19 | 22576813 |
| LFQ intensity B2-20 | 219864429 | LFQ intensity A2-20 | 261914056 |
