# Supplementary material for: AARS2 as a novel biomarker for prognosis and its molecular characterization in pan‐cancer
Source: Cancer Med. 2023 Nov 21;12(23):21531–44. doi: 10.1002/cam4.6682 (PMC10726843; doi:10.1002/cam4.6682)
Supplement: Supplementary file 5 — Table S3 [file CAM4-12-21531-s005.docx]

| **Table S3. The detailed sequences of siRNAs in this study.** | | |
| --- | --- | --- |
| **Name** | **Sense** | **Antisense** |
| Negative control | UUCUCCGAACGUGUCACGUTT | ACGUGACACGUUCGGAGAATT |
| Si-AARS2-1 | CCAGUUCAAGCCAAUCUUUTT | AAAGAUUGGCUUGAACUGGTT |
| Si-AARS2-2 | GGGAGAUGCUUAUCCAGAATT | UUCUGGAUAAGCAUCUCCCTT |
| Si-AARS2-3 | GCUUGAUGUCCAUGCGCUUTT | AAGCGCAUGGACAUCAAGCTT |
